# Days gained response discriminates treatment response in patients with recurrent glioblastoma receiving bevacizumab-based therapies

**DOI:** 10.1093/noajnl/vdaa085

**Published:** 2020-07-09

**Authors:** Kyle W Singleton, Alyx B Porter, Leland S Hu, Sandra K Johnston, Kamila M Bond, Cassandra R Rickertsen, Gustavo De Leon, Scott A Whitmire, Kamala R Clark-Swanson, Maciej M Mrugala, Kristin R Swanson

**Affiliations:** 1 Mathematical NeuroOncology Lab, Precision NeuroTherapeutics Innovation Program, Mayo Clinic, Phoenix, Arizona; 2 Division of Neuro-Oncology, Department of Neurology, Mayo Clinic, Phoenix, AZ; 3 Department of Radiology, Mayo Clinic, Phoenix, Arizona; 4 Department of Radiology, University of Washington, Seattle, Washington; 5 Mayo Clinic Alix School of Medicine, Mayo Clinic, Rochester, Minnesota; 6 Department of Neurosurgery, Mayo Clinic, Phoenix, Arizona

**Keywords:** bevacizumab, combination chemotherapy, glioblastoma, personalized medicine, response evaluation

## Abstract

**Background:**

Accurate assessments of patient response to therapy are a critical component of personalized medicine. In glioblastoma (GBM), the most aggressive form of brain cancer, tumor growth dynamics are heterogenous across patients, complicating assessment of treatment response. This study aimed to analyze days gained (DG), a burgeoning model-based dynamic metric, for response assessment in patients with recurrent GBM who received bevacizumab-based therapies.

**Methods:**

DG response scores were calculated using volumetric tumor segmentations for patients receiving bevacizumab with and without concurrent cytotoxic therapy (*N* = 62). Kaplan–Meier and Cox proportional hazards analyses were implemented to examine DG prognostic relationship to overall (OS) and progression-free survival (PFS) from the onset of treatment for recurrent GBM.

**Results:**

In patients receiving concurrent bevacizumab and cytotoxic therapy, Kaplan–Meier analysis showed significant differences in OS and PFS at DG cutoffs consistent with previously identified values from newly diagnosed GBM using T1-weighted gadolinium-enhanced magnetic resonance imaging (T1Gd). DG scores for bevacizumab monotherapy patients only approached significance for PFS. Cox regression showed that increases of 25 DG on T1Gd imaging were significantly associated with a 12.5% reduction in OS hazard for concurrent therapy patients and a 4.4% reduction in PFS hazard for bevacizumab monotherapy patients.

**Conclusion:**

DG has significant meaning in recurrent therapy as a metric of treatment response, even in the context of anti-angiogenic therapies. This provides further evidence supporting the use of DG as an adjunct response metric that quantitatively connects treatment response and clinical outcomes.

**Key Points**

- Days gained (DG) response metric applicable for glioblastoma (GBM) patients with anti-angiogenic therapy.
- Significant association of survival outcomes with DG metric in recurrent GBM.
- DG is a consistent response metric across primary and recurrent GBM patients.

**Importance of the Study**

In response to the dire need for methods to detect clinically meaningful responses to treatment in individual patients, the Response Assessment in Neuro-Oncology (RANO) working group has proposed new investigations of advanced imaging and volumetric measurements to improve the assessment of treatment response in glioblastoma (GBM). A model-based approach using volumetric tumor measurements that captures the dynamics of tumor growth, such as Days Gained (DG), can account for patient-to-patient heterogeneity to provide additional insight into response to therapy. Our study indicates DG provides significant insight into treatment response for overall (OS) and progression-free survival (PFS) even in the challenging context of anti-angiogenic therapy. These findings were consistent with prior DG analysis, indicating that DG scores can provide a stable marker across treatment settings. Prospective evaluation of the DG metric is required to further determine the role a growth-kinetics based model can provide for evaluating OS and PFS.

## Introduction

In the era of precision-based medicine, clinicians strive to understand the unique evolution of disease in individual patients to provide the most effective care. Evaluation of treatment response and outcomes is of particular importance in clinical trials for comparing the effectiveness of novel therapies to the current standard of care. In the clinical setting, these same measurements of treatment response would ideally help clinicians assess tumor status as early as possible, allowing informed decisions for adjusting therapies. In patients with glioblastoma (GBM), the most common and aggressive form of glioma, the management of recurrent GBM has posed significant challenges with limited success in clinical trials for the last several decades. Despite aggressive therapy, the highly invasive and dynamic nature of GBM inevitably leads to tumor recurrence, usually as defined by existing response metrics. At recurrence, previously useful therapies are often presumed ineffective and patients are transitioned to another therapy. To address the challenge of treatment appraisal in the setting of recurrent GBM, we evaluated patients receiving bevacizumab with and without concurrent cytotoxic therapies using a personalized model-based response metric, days gained (DG), that utilizes volumetric image measurements to capture differing tumor growth dynamics between patients.

GBM patients’ typical length of survival from the time of diagnosis is less than 2 years^1,2^ for patients over 70 or following tumor recurrence.^3^ Standard-of-care treatment consists of oral chemotherapy with alkylating agents and concomitant radiation therapy for a total of 6 weeks; adjuvant chemotherapy is then recommended for 6–12 months in the absence of disease progression or toxic side effects.^4^ Recurrent therapy commonly includes the use of bevacizumab, a humanized monoclonal antibody that targets vascular endothelial growth factor to inhibit angiogenesis, thereby reducing the tumor’s vascular supply. Reports from the AV37018g and NCI 06-C-0046E trials of bevacizumab for recurrent GBM were promising for improved progression-free survival (PFS), but improvements were not observed for overall survival (OS).^5,6^ These trials resulted in FDA approval of bevacizumab as a single-agent treatment for recurrent GBM. While subsequent clinical trials have not provided conclusive evidence that bevacizumab improves OS, they have solidified the impact of bevacizumab on PFS and clinicians continue to use it both as a monotherapy and in combination with cytotoxic agents.^7^

As bevacizumab inhibits neoangiogenesis and normalizes the blood–brain barrier within the tumor, gadolinium extravasation is diminished and contrast enhancement on T1-weighted imaging diminishes. These effects can be visualized as early as 1–2 days after therapy and can persist for the duration of bevacizumab administration. As such, assessing the efficacy of bevacizumab with imaging has proven difficult due to this “pseudo-response” effect, where imaging response may reflect anti-angiogenic response rather than significant cytoreduction in tumor cell burden. These effects may also be important to consider in T2-weighted sequences, as in one study, it was noted that as many as 37% of patients receiving bevacizumab had tumor recurrence defined specifically by T2/FLAIR changes.^8^

Current imaging-based treatment response metrics in cancer utilize one-dimensional (RECIST) or two-dimensional (Macdonald, Response Assessment in Neuro-Oncology [RANO]) measurements of tumor abnormality.^9–13^ These measurements capture only a portion of the total abnormality seen on MRI and do not represent the entire scope of disease for each patient. As a result, current metrics are limited in their ability to describe patient-specific differences in tumor size and growth in GBM and have shown little success in predicting patient outcome.^13,14^ In recent years, the RANO working group has provided useful criteria for standardizing the assessment of the response of high-grade gliomas to treatment, but there continues to be a discussion on how to expand on these guidelines by considering data from advanced imaging, digital subtraction maps, and volumetric measurements.^12,13^

A number of mathematical models have been previously investigated for the purpose of simulating tumor growth kinetics using volumetric tumor measurements from clinical imaging data.^15–20^ The kinetics of tumor growth have been shown to vary greatly across patients due to the heterogeneous nature of GBM. Consequently, developing response metrics that account for tumor kinetics can aid in the understanding of tumor aggressiveness that have not been taken into account with current metrics.^21,22^ These mathematical models generate patient-specific untreated virtual controls (UVCs) that can be used to estimate anticipated growth at future time points. UVC tumor size can then be used for comparison against actual tumor growth for each patient. Utilizing this model-based approach, a patient-specific metric, DG, was defined as the degree to which a given treatment deflected tumor growth, measured in days.^23^ DG was found to be prognostic for both OS and PFS in the context of first-line, standard-of-care radiotherapy in newly diagnosed GBM patients.^23^ DG was further assessed in the first-line radiotherapy setting for sensitivity to three UVC tumor models, applying different levels of computational complexity (four-dimensional anatomic, four-dimensional spherical, and linear).^24^ In each case, DG remained prognostic for OS and PFS, indicating that simplified versions of the UVC were appropriate to reduce computational time and make the DG metric more readily accessible to clinicians. In addition, this work found that DG was able to discriminate progression versus pseudoprogression following radiotherapy. DG was also applied in a novel early phase gene therapy clinical trial using autologous gene-modified hematopoietic stem cells, comparing DG scores with standard-of-care therapy.^25^ DG values were higher for patients undergoing the novel therapy indicating this treatment caused a greater deflection of tumor growth than standard care.

Based upon these prior successes, we attempt to further elucidate the capability of DG in the recurrent setting where treatments begin to vary and can be given in combination. In particular, the incorporation of patient-specific kinetics into metrics of response can allow for earlier predictions of treatment response as seen in upfront analysis. Earlier response detection can minimize imaging follow up and is critical in recurrent care where OS and PFS times drop significantly. As noted above, bevacizumab use for the treatment of GBM has been widespread since FDA approval. Consequently, bevacizumab is frequently given with other therapies, but the pseudo-response effect of bevacizumab can impair the assessment of response. In this work, we investigate a cohort of patients who either received bevacizumab as monotherapy or in combination with cytotoxic therapies using the DG response metric to evaluate discrimination of OS and PFS outcomes.

## Methods

Following institutional review board approval, we identified 67 patients diagnosed with recurrent GBM who received bevacizumab therapy with or without concurrent cytotoxic therapy from our multi-institutional clinical research database. Patients were treated between 2006 and 2016 across six institutions, with MRIs obtained from both 1.5T and 3T GE, Siemens, and Philips scanners. Patients were required to have T1-weighted gadolinium-enhanced (T1Gd) and T2-weighted fluid-attenuated inversion recovery (FLAIR) magnetic resonance images on two pretreatment and one posttreatment date from the start of bevacizumab therapy for inclusion. MRI protocols varied due to the multi-institutional nature of the cohort with the following most common settings: T1Gd (repetition time [TR]/echo time [TE] = 400 ms/10 ms; voxel size = 0.45 × 0.45 × 5 mm^3^); FLAIR (TR/TE/inversion time [TI] = 11,000 ms/140 ms/2800 ms; voxel size = 0.45 × 0.45 × 5 mm^3^). We reviewed the treatments of each recurrent GBM patient to determine what concurrent cytotoxic treatment, if any, were given alongside bevacizumab therapy. Patients in our dataset received concurrent carboplatin, CCNU (lomustine), BCNU (carmustine), Gliadel Wafers, or CPT-11 (irinotecan) with bevacizumab. Carboplatin was the most common concurrent cytotoxic therapy administered (*N* = 25). Patients were analyzed as a complete set of bevacizumab-based therapies and were also analyzed as subgroups of bevacizumab monotherapy (BevAlone, *N* = 24) and bevacizumab plus cytotoxic agent (BevCyto, *N* = 38). Two patients in the BevAlone group were treated during a potential pseudoprogression period within 12 weeks of completion of radiation therapy. All other patients were treated outside the pseudoprogression window and no patients received bevacizumab-based therapy overlapping with adjuvant temozolomide. Genetic records were sparse in the available cohort, with the less than 15% of cases with known status for markers such as MGMT, IDH1, and 1p19q status. For example, we found two MGMT methylated and six MGMT unmethylated patients were indicated in our cohort. No patients had multiple genetic determinants as noted by the revised WHO criteria.^26^ Due to this sparsity in the data, we did not analyze genetic components as part of this analysis.

**Figure 1.**
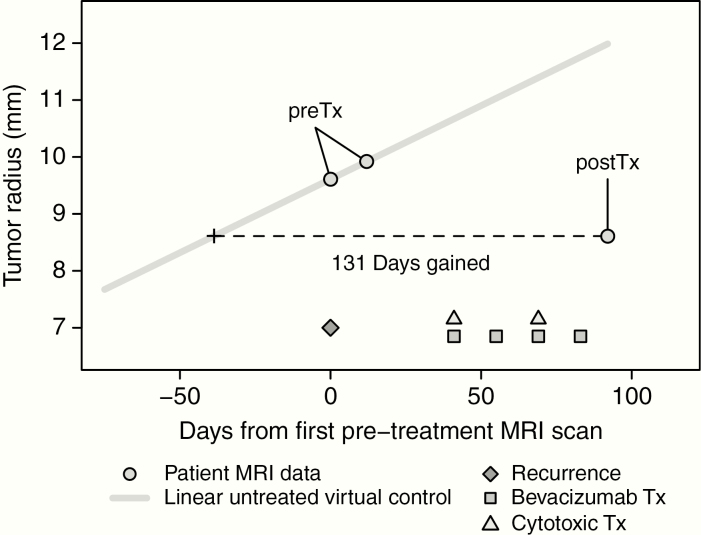
Example of computing the DG metric for a BevCyto case in the recurrent setting, adapted from previously published linear DG methodology.^23,24^ Pretreatment (preTx, first two circles) and posttreatment (postTx, third circle) MRIs are segmented for each contrast (ie T1Gd or FLAIR). A patient-specific untreated virtual control can be calculated with preTx data (grey line). DG scores are then calculated by comparing postTx tumor size with the untreated growth rate to determine the amount of time gained or lost depending on tumor change during therapy. Timing of clinical events relative to imaging is shown at the bottom of the figure. Patients in this study received bevacizumab with or without concurrent cytotoxic therapy and were not required to have a specific number of cycles or complete therapy prior to first posttreatment imaging.

Patient response to therapy was evaluated using our previously described DG response metric.^23,24^ Briefly, DG is computed by comparing posttreatment volumetric tumor size against a patient-specific prediction of untreated tumor size over the period of time between imaging studies. In this article, we calculate DG scores using the linear DG method, described by Neal.^24^ The linear DG method, comparable to more complex UVC models, is easier to compute and generalize for the use of DG across upfront and recurrent treatment evaluations. DG scores for both T1Gd and FLAIR MRIs were considered to look into the potential predictive ability of enhancing and nonenhancing regions of tumor abnormality, similar to current evaluations using the RANO criteria. All DG methods require the use of two MRI time points prior to the start of therapy to define tumor growth characteristics and calculate patient-specific predictions of tumor growth. For this study, treatment is defined based upon the start of bevacizumab therapy for treatment of recurrent tumor. Pretreatment images are then defined as the two MRI scan dates preceding bevacizumab infusion and posttreatment images as the first available scan date following infusion. Patients were not required to have a defined time interval between imaging dates and were not required to be off therapy during their first posttreatment MRI. This relative timing of treatment and imaging is shown in an example in Figure 1.

Volumetric tumor segmentations were performed for each time point (2× pre- and 1× posttreatment) and image subtype (T1Gd, FLAIR) in our cohort using a semi-automated segmentation process by one or more independent image analysts trained in MRI segmentation. All volumetric segmentations used for our analysis included necrotic regions as part of the total volume of tumor abnormality. This was done as the underlying assumptions of the DG model result in linear radial growth of the entire abnormality, not the parts separately. Spherically equivalent radial tumor sizes of the tumor abnormality were estimated and patient-specific tumor growth velocities were calculated using radial tumor sizes from pretreatment MRIs. These growth characteristics can then be used to provide untreated growth predictions over time. DG scores are then obtained by comparing the predictions of untreated tumor growth to actual posttreatment tumor size over the time interval between second pretreatment MRI and posttreatment MRI. If an additional cytotoxic therapy was given in the interval between second pre- and first posttreatment imaging, the case was defined as a BevCyto case (bevacizumab plus cytotoxic agent). Otherwise, the case was defined as a BevAlone case (bevacizumab monotherapy).

For each treatment group and imaging sequence type, we performed Kaplan–Meier analysis as well as univariate and multivariate Cox proportional hazards models for PFS and OS. Kaplan–Meier analysis was first performed on the full dataset using previous cutoffs from DG evaluation in patients receiving radiotherapy for newly diagnosed GBM.^24^ This method was used to evaluate if prior DG findings would generalize across treatment settings. In addition, these previous cutoffs were applied in the treatment subgroups and compared to median DG cutoffs from the recurrent study patients. Each evaluation cutoff was used to define “High” and “Low” DG response groups with DG scores above and below the cutoff of interest in the different Kaplan–Meier analysis. Univariate Cox proportional hazards were performed for DG and multivariate Cox analysis for DG, age at the start of therapy, and patient sex. Kaplan–Meier and Cox proportional hazards analysis were performed in R (v3.6.1) using the *survival* and *survminer* packages.^27,28^ OS and PFS were defined as the time interval between the start of the patient’s recurrent therapy to the date of death or date of progression, respectively, as documented in the clinical chart. Patients were censored at the last known date of follow-up if the outcome in question was not available.

## Results

### Patient Cohort

A total of 62 patients with recurrent GBM met the criteria for inclusion in this study. Five cases were removed due to a lack of required imaging time points. Of these patients, 24 received bevacizumab monotherapy (BevAlone) and 38 received bevacizumab with concurrent cytotoxic therapy (BevCyto). DG scores were calculable from T1Gd imaging (DG_T1Gd_) for all 62 patients. Nine cases with negative FLAIR pretreatment tumor growth velocities were excluded, yielding 53 patients with calculable DG from FLAIR imaging (DG_FLAIR_), as DG scores require positive tumor growth values for computation. A summary of patient demographics and calculated DG scores for T1Gd and FLAIR imaging are provided in Table 1. Negative DG scores occur when posttreatment tumor size is larger than the anticipated growth shown using the UVC, implying a faster than expected growth rate.

**Table 1.** Demographics of patients with recurrent GBM by treatment group evaluated with DG scores

	BevAlone	BevCyto
*N*	24	38
Sex		
Male	14 (58%)	28 (74%)
Female	10 (42%)	10 (26%)
Age (start of treatment)		
Mean (range)	54.8 (20–77)	58.0 (22–79)
DG T1GD		
Median	134.4	95.9
Range	(−852.7, 648.7)	(−66.76, 702)
DG FLAIR		
Median	121.3	57.8
Range	(−643.3, 630.7)	(−921.4, 390.7)

Patients received either bevacizumab monotherapy (BevAlone) or bevacizumab concurrent with a cytotoxic therapy (BevCyto).

### Prior Newly Diagnosed DG Cutoffs Remain Significant in Bevacizumab-Treated Recurrent Patients

**Figure 2.**
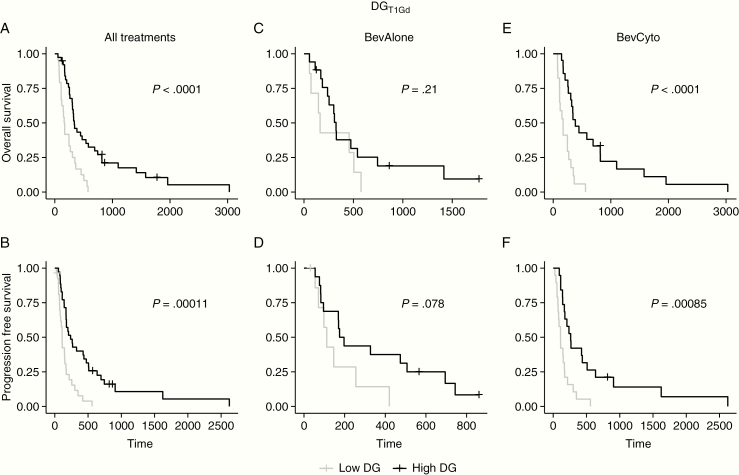
Kaplan–Meier analysis of DG_T1Gd_ using previously identified optimal DG_T1Gd_ cutoffs^24^ in the complete cohort (first column, A–B) and subanalysis by treatment group (second and third columns, C–F) for OS and PFS. Prior cutoffs significantly discriminated survivor groups in the recurrent setting for OS and PFS. In further subanalysis by treatment, prior cutoffs significantly discriminated survivor groups for BevCyto patients (E–F), but not for BevAlone patients (C–D). High and Low DG groups were set based on the assigned cutoffs reported in Table 2.

In prior analysis of newly diagnosed GBMs receiving standard-of-care therapies, a set of optimal DG scores for T1Gd imaging were identified with a statistically significant survival benefit.^23,24^ The optimal prior threshold for discrimination of OS was 78 DG and for PFS was 93 DG using the linear DG model. These thresholds were applied to the complete cohort of 62 recurrent GBM patients who received bevacizumab-based therapy. High and Low DG response groups were evaluated based on patients with DG scores above or below the given cutoff for OS and PFS. For DG_T1Gd_ scores, each DG cutoff from the newly diagnosed setting continued to successfully distinguish OS and PFS in the recurrent setting (Figure 2A and B, log-rank *P* values < .0001). However, the previous cutoffs were not significant for DG_FLAIR_ scores (Supplementary Figure S2), likely because the cutoffs from the previous newly diagnosed analysis were defined using T1Gd imaging of the tumor abnormality. In addition, median cutoffs from the recurrent population were applied to explore any potential changes in significance due to the difference between the newly diagnosed and recurrent populations. While the median cutoffs varied from the prior cutoffs, discrimination for OS and PFS was consistent with strong significance (Supplementary Figure S1A and B, log-rank *P* values < .00114).

**Table 2.** Efficacy of median recurrent cutoff and previously published newly diagnosed cutoffs (Neal et al.^24^) for discriminating OS and PFS across all treatment groups

		Treatment group	Median cutoffs	*P* value	Neal cutoffs	*P* value
DG T1Gd	OS	All treatments	106.7	.00114	78	9.25E−05
		BevAlone	134.4	.18	78	.21
		BevCyto	95.9	.00036	78	9.25E−05
	PFS	All treatments	106.7	.00063	93	.00011
		BevAlone	134.4	.059	93	.078
		BevCyto	95.9	.00085	93	.00085
DG FLAIR	OS	All treatments	74.4	.80	78	.54
		BevAlone	121.3	.74	78	.80
		BevCyto	57.8	.55	78	.33
	PFS	All treatments	74.4	.51	93	.25
		BevAlone	121.3	.91	93	.62
		BevCyto	57.8	.10	93	.08

Analysis was performed for both T1Gd- and FLAIR-based DG scores. Significant log-rank test *P* values underlined.

### Subanalysis of Patients Treated with Cytotoxic Therapies in Combination with Bevacizumab

To further consider the influence of therapy on the use of generalized versus cohort-specific DG cutoffs, prior and median DG score cutoffs were applied in a subanalysis of patients split by therapy. This provided median cutoffs specific to each treatment subgroup, MRI contrast, and survival evaluation. Newly diagnosed cutoffs varied for OS and PFS, but not by MRI contrast or treatment. Comparing across subgroups provides more detail on the capability of DG scores to differentiate responses in varied treatment scenarios and the differences in discrimination provided by volumetric inputs of T1Gd and FLAIR MRI contrasts. All applied cutoffs and log-rank significance are presented in Table 2. Kaplan–Meier curves are provided in Figure 2 for the DG_T1Gd_ prior cutoff analysis and in supplementary material for other cutoffs (Supplement Figures S1–S3). Each prior and median cutoff was used to define High DG and Low DG response groups for the respective Kaplan–Meier analysis. Prior and Median DG_T1Gd_ scores for BevCyto patients were highly significant for both OS and PFS in Kaplan–Meier analysis log-rank tests (Table 2, Figure 2, and Supplementary Figure 1E–F). In contrast, the BevAlone group was not significantly discriminated in Kaplan–Meier analysis using either the median or prior cutoffs (Table 2, Figure 2 and Supplementary Figure 1C–D). In addition, neither BevAlone nor BevCyto showed a significant difference between the high and low DG cutoffs for any threshold when using DG_FLAIR_ scores (Table 2, Supplement Figures S2 and S3C–F).

### Robustness of DG as a Predictor of Survival

**Figure 3.**
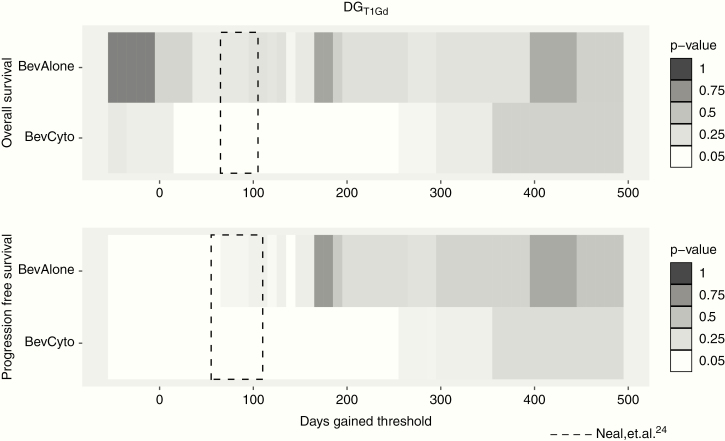
Iterative Kaplan–Meier significance of DG_T1Gd_ thresholds for OS and PFS for each therapy group (white: log-rank *P ≤* .05, greys: *P* > .05). Significant cytotoxic therapy thresholds show substantial overlap with DG thresholds from newly diagnosed treatment analysis (dashed box^24^).

Using an iterative Kaplan–Meier analysis, we further explored the range of DG thresholds that discriminate for OS and PFS in each therapy group. Figure 3 illustrates these results for OS and PFS across both treatment groups using DG_T1Gd_ thresholds. DG_T1Gd_ thresholds that reached significance were broadly seen in a range between 20 and 250 DG_T1Gd_ for OS in BevCyto patients. This range was consistent for PFS. In addition, these significant DG ranges overlap with previous findings for DG cutoffs in newly diagnosed patients receiving upfront therapy, where significant DG thresholds ranged from 65 to 105 for OS and 55 to 110 for PFS (Figure 3, dashed box).^24^ In the BevAlone group, no significant DG_T1Gd_ thresholds were seen for OS in BevAlone patients. A few significant thresholds for BevAlone PFS were observed around 60 and 140 DG for T1Gd and FLAIR analysis, but other intermediate thresholds were not significant. Thresholds for DG_FLAIR_ analysis were not significant in most cases, although a few significant thresholds were observed for PFS in the BevCyto therapy group (Supplementary Figure S4).

### Cox Proportional Hazard Analysis of DG

In Cox proportional hazards regression analysis, DG_T1GD_ was a significant predictor of OS for the concurrent therapy groups in univariate analysis and after controlling for patient age and sex in multivariate analysis (Table 3). In the BevCyto group, for example, patients had a 12.5% reduction in the chance of death for every 25 DG. Treatment with bevacizumab monotherapy, BevAlone, was not a significant predictor of patient OS, but was significant for PFS (Table 3). Patients in the BevAlone group saw a 4.4% reduction in the chance of progression for every 25 DG. Similar trends were seen for PFS in the BevCyto group, with a reduction of 12.3% for concurrent therapy. Male sex was also a significant predictor of decreased survival in some models (Table 3). DG_FLAIR_ scores were not significant as a predictor of patient OS or PFS in either treatment group (Supplementary Table S1).

**Table 3.** Cox proportional hazards regression analysis of OS and PFS using continuous DG_T1Gd_ scores, patient age at the start of treatment, and patient sex

		Model	Variable	HR	95% CI	*P* value
OS	BevAlone	Univariate	25 DG T1Gd	0.985	[0.954, 1.016]	.333
		Multivariate	25 DG T1Gd	0.988	[0.950, 1.027]	.543
			Age	1.017	[0.973, 1.062]	.459
			Sex (M)	3.618	[1.137, 11.509]	.029
	BevCyto	Univariate	25 DG T1Gd	0.879	[0.809, 0.955]	.002
		Multivariate	25 DG T1Gd	0.875	[0.802, 0.956]	.003
			Age	1.008	[0.981, 1.037]	.559
			Sex (M)	1.859	[0.840, 4.118]	.126
PFS	BevAlone	Univariate	25 DG T1Gd	0.960	[0.925, 0.997]	.034
		Multivariate	25 DG T1Gd	0.956	[0.917, 0.998]	.038
			Age	1.017	[0.972, 1.064]	.456
			Sex (M)	3.995	[1.306, 12.217]	.015
	BevCyto	Univariate	25 DG T1Gd	0.872	[0.798, 0.953]	.002
		Multivariate	25 DG T1Gd	0.877	[0.801, 0.960]	.004
			Age	1.007	[0.981, 1.033]	.620
			Sex (M)	1.610	[0.726, 3.567]	.241

Significant *P* values underlined.

## Discussion

In this article, we examined the use of a patient-specific response metric, DG, for the discrimination of OS and PFS in patients with recurrent GBM who received bevacizumab with or without concurrent cytotoxic therapy. Patients were grouped by therapy to examine the possible pseudo-responsive effects from bevacizumab on response scoring using DG. Our results show that DG scores significantly discriminate OS and PFS among patients with recurrent tumor receiving concurrent cytotoxic therapy with bevacizumab (BevCyto) using measurements of enhancing tumor. Most importantly, this finding indicates DG is useful for discrimination even in the challenging context of bevacizumab-based therapies that are known to significantly impact imaging changes but with unclear benefit in OS outcomes. Thus, DG can serve as a stable marker of response for patients receiving cytotoxic therapies across newly diagnosed and recurrent settings, providing a patient-specific metric of response that can add context to clinical decision-making with regard to treatment course.

A pooled analysis of DG for all cases from our treatment cohorts was significant with regard to discriminating outcomes, however, the majority of discrimination effect was related to patients receiving concurrent cytotoxic therapy with bevacizumab. Concurrent therapy patients (BevCyto) were able to be discriminated into survival groups using DG_T1Gd_ scores with high significance, but bevacizumab monotherapy (BevAlone) only denoted a detectable benefit for PFS (Table 2, Figure 2). Similar results were seen when evaluating across DG cutoffs using an interactive Kaplan–Meier analysis where BevCyto patients had a number of valid response cutoffs, but BevAlone patients had very few (Figure 3). These results indicate that DG scores are correlated with survival changes (ie decreases in DG scores correlate with decreased survival) when cytotoxic agents are used but not when anti-angiogenic therapy is given alone. For cases including concurrent bevacizumab therapy, these findings also indicate that treatment response is detectable using DG scores in the presence of potential pseudo-response effects, making DG useful in both newly diagnosed^24^ and recurrent treatment scenarios. This stands in contrast to clinical trials that have found no OS benefit to combination bevacizumab therapy. For example, a study of progressive GBM compared lomustine, a common therapy at first progression, with lomustine in combination with bevacizumab.^29^ The addition of bevacizumab in the trial conferred a prolongation of PFS without a significant OS advantage. Our findings indicate there may be subpopulations of patients who benefit from combination therapy that are difficult to detect. DG appears to be able to help detect these individual patients, but this requires further study. Cox proportional hazards modeling further supports DG as a significant indicator of OS and PFS in assessing recurrent GBM response in the presence of bevacizumab. Patients receiving concurrent therapy in this work saw a significant reduction in hazard of 12.5% per increase of 25 DG_T1Gd_ for OS (Table 3). A similar reduction in hazard of PFS was seen for increasing DG in these patients. A more modest 4.4% hazard reduction per 25 DG_T1Gd_ for PFS was also seen for the bevacizumab monotherapy group (Table 3).

Notably, our analysis of DG values calculated from FLAIR images did not show a strong relationship with OS or PFS for any treatment group. Measuring T2/FLAIR abnormality can be difficult and has been noted by the RANO working group as a reason why objective criteria have not been added for nonenhancing image assessments in the RANO criteria.^12^ A number of cases in the FLAIR evaluation showed negative pretreatment tumor growth and were excluded in analysis. In addition, while bevacizumab treatment is routinely associated with significant changes in the extent of abnormality on T1Gd, the effect on T2/FLAIR sequences is more heterogeneous. As a result, we expect that there may be more subtle differences in imaging changes in the T2/FLAIR sequences. While our FLAIR findings were not significant, we believe additional analysis with T2/FLAIR imaging is still warranted as other studies have found a difference in OS and PFS between patients with differing radiologic progression patterns.^8,30^ In addition, as with assessment with the RANO criteria, assessment at future imaging timepoints after more cycles of bevacizumab therapy may be required to detect these differences between patients.

We acknowledge the innate limitations of performing a retrospective analysis of patients. Treatment options for recurrent GBM have varied significantly over time with attempts to improve patient outcomes, yet there is no defined standard-of-care for this recurrent context. We combined patients who received a variety of different concurrent treatments to serve as a cytotoxic treatment group, but the signal may be confounded by additional surgery or lack of efficacy of some treatments. However, our findings indicate that these effects do not limit DG ability to discriminate responders overall. In addition, the retrospective nature of our cohort limited the ability to collect other clinical parameters that can influence patient outcomes. For this article, we evaluated available features such as age and sex. However, we had significant missing data for performance metrics (KPS) and genetic markers (MGMT, IDH-1), that did not allow for robust analysis or multiple imputation with these items. Similarly, the retrospective approach of this work limited our sample size. Given the heterogeneity in treatment profiles of recurrent patients and frequently nonstandardized, varying intervals of scheduled MRI scans, we were limited in the number of cases we were able to include in our analysis.

Another potential limitation to our method is the variability in the inputs used to calculate DG scores. Sources of variability include inter- and intra-observer error in volumetric tumor segmentation, the time between the collection of images, and the underlying tumor growth dynamics. In recent work, we explored the impact of different types of data uncertainty on the robustness of DG scores.^31^ Broadly, we found that the DG response metric was robust to reasonable segmentation error, but was more sensitive to the time between the imaging time points and the underlying growth dynamics. The metric was more robust when there was a longer time delay between the imaging time points and for slower-growing tumors. Thus, while we have not directly assessed the degree of sensitivity in this cohort, both these sources of potential uncertainty can be estimated at the time of calculation and accounted for when either designing a study protocol or when used in clinical decision making.

In conclusion, our study indicates that DG, an individualized metric of response to therapy, was able to discriminate OS and PFS for recurrent GBM patients receiving cytotoxic therapies in combination with bevacizumab, but could only discriminate PFS for those receiving bevacizumab alone. This finding is consistent with the growing literature that bevacizumab monotherapy does not impact OS. The discriminative power of DG for cytotoxic therapy using T1Gd MRI does not appear to be negatively affected by the administration of concurrent bevacizumab. Significant DG thresholds in the recurrent setting were consistent with prior newly diagnosed DG findings, as thresholds from prior studies of standard-of-care therapy were applicable to the recurrent setting, providing evidence of stability in DG predictions. While GBM ultimately remains fatal in all patients, being able to prognosticate based on the relationship between volumetric changes and a patient’s tumor-specific growth-kinetics provides clinical value. Our method may serve as a useful adjunct to the RANO criteria which are used to classify responders from nonresponders to therapy. DG offers a method to incorporate tumor individuality with regard to the dynamics of growth prior to treatment. Thus, assessing patients with DG can provide additional context to overall tumor size and new opportunities to adjust followup times for each patient, particularly those with low DG, where response appears to be limited. Translating treatment and followup schedules based upon patient-specific context may help clinicians gain traction towards the goal of connecting response to clinical benefit, as measured in terms of outcomes like OS.

## Supplementary Material

vdaa085_suppl_Supplementary_Material

## Notes

**Authorship statement**. Study conception and design was done by A.B.P., L.S.H., M.M.M., and K.R.S. Data collection by K.W.S., S.K.J., C.R.R., G.L., S.A.W., and K.R.C.S. Data analysis and interpretation by K.W.S., S.K.J., K.M.B., A.B.P., and K.R.S. Manuscript preparation by K.W.S., K.M.B., C.R.R., S.K.J., A.B.P., and K.R.S. All authors reviewed and approved the final manuscript.
