# Supplementary material for: Days gained response discriminates treatment response in patients with recurrent glioblastoma receiving bevacizumab-based therapies
Source: Neurooncol Adv. 2020 Jul 9;2(1):vdaa085. doi: 10.1093/noajnl/vdaa085 (PMC7447137; doi:10.1093/noajnl/vdaa085)
Supplement: vdaa085_suppl_Supplementary_Material [file vdaa085_suppl_supplementary_material.docx]

**Supplemental Figures and Tables**

***Figure SF1:*** *Kaplan-Meier analysis of* DG_T1Gd_ *using median DG_T1Gd_ cutoffs from the recurrent cohort in the complete cohort (first column, A-B) and subanalysis by treatment group (second and third columns, C-F) for overall survival and progression free survival. Newly diagnosed cutoffs significantly discriminated survivor groups in the recurrent setting for OS and PFS overall, matching with findings using prior newly diagnosed cutoffs. Median cutoffs were also significant in the subanalysis for BevCyto patients, discriminating survivor groups (E-F), but not for BevAlone patients (*C-D)*. High and Low DG groups were set based on the assigned cutoffs reported in Table 2.*

**
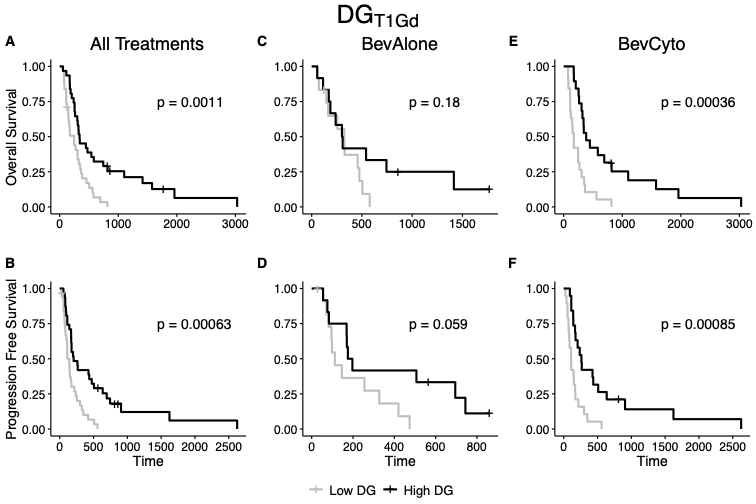
**

***Figure SF2:*** *Kaplan-Meier analysis of* DG_FLAIR_ *using previously identified optimal DG_T1Gd_ cutoffs*[*^24^*](https://paperpile.com/c/1OHGbT/k61m) *in the complete cohort (first column, A-B) and subanalysis by treatment group (second and third columns, C-F) for overall survival and progression free survival. No significant discrimination between groups was observed in either the full cohort or subanalysis. High and Low DG groups were set based on the assigned cutoffs reported in Table 2.*

**
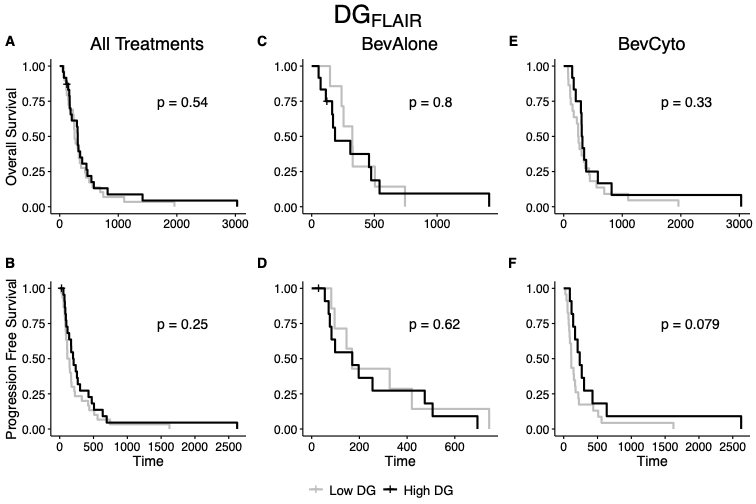
**

***Figure SF3:*** *Kaplan-Meier analysis of* DG_FLAIR_ *using median DG_FLAIR_ cutoffs from the recurrent cohort in the complete cohort (first column, A-B) and subanalysis by treatment group (second and third columns, C-F) for overall survival and progression free survival. No significant discrimination between groups was observed in either the full cohort or subanalysis. High and Low DG groups were set based on the assigned cutoffs reported in Table 2.*

**
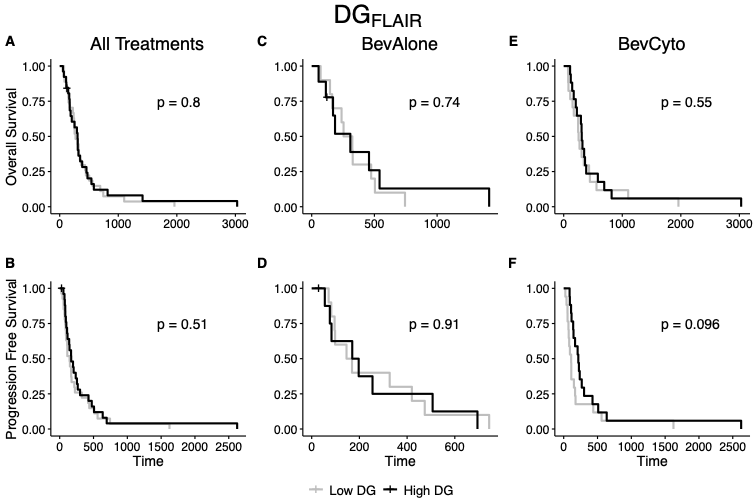
**

**Figure SF4**: *Iterative Kaplan-Meier significance of* DG_FLAIR_ *thresholds for overall survival and progression free survival for each therapy group (white: p<=0.05, greys: p>0.05). Very few thresholds overlap with Days Gained thresholds from prior adjuvant treatment analysis (dashed box*[*^24^*](https://paperpile.com/c/1OHGbT/k61m)*) based on T1Gd imaging.*

**
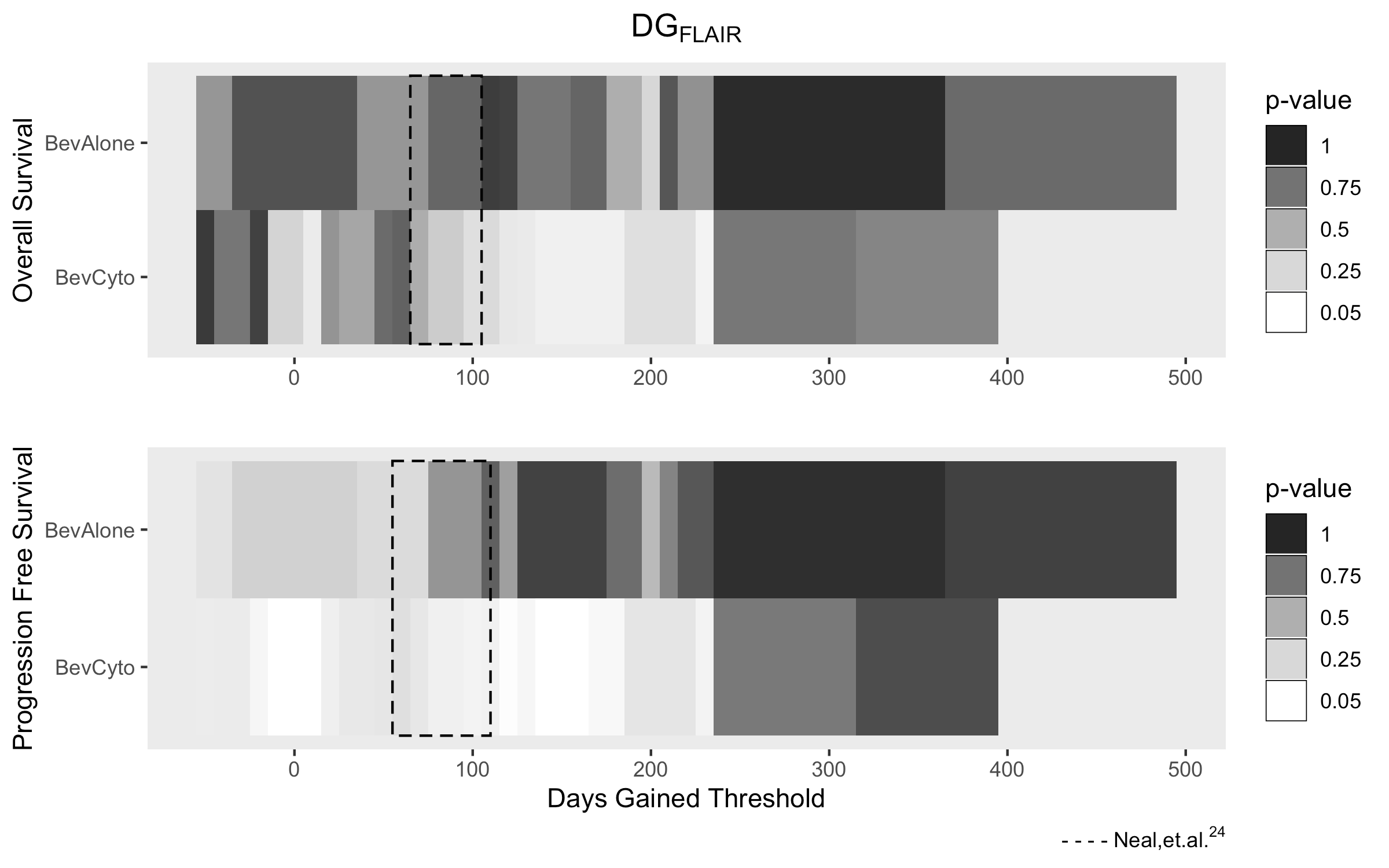
**

**Table ST1:** *Cox proportional hazards regression analysis of overall survival and progression free survival using continuous DG_FLAIR_ scores, patient age at start of treatment, and patient sex. No significant p-values were observed.*

|  | | Model | Variable | HR | 95% CI | p-value |
| --- | --- | --- | --- | --- | --- | --- |
| Overall Survival | BevAlone | Univariate | 25 DG FLAIR | 0.993 | [0.937, 1.053] | 0.827 |
|  |  | Multivariate | 25 DG FLAIR | 0.983 | [0.929, 1.039] | 0.545 |
|  |  |  | Age | 1.011 | [0.968, 1.056] | 0.619 |
|  |  |  | Sex (M) | 2.781 | [0.911, 8.495] | 0.073 |
|  | BevCyto | Univariate | 25 DG FLAIR | 0.996 | [0.963, 1.030] | 0.797 |
|  |  | Multivariate | 25 DG FLAIR | 0.998 | [0.962, 1.035] | 0.913 |
|  |  |  | Age | 1.018 | [0.991, 1.046] | 0.188 |
|  |  |  | Sex (M) | 1.728 | [0.767, 3.893] | 0.187 |
| Progression Free Survival | BevAlone | Univariate | 25 DG FLAIR | 1.011 | [0.959, 1.066] | 0.693 |
|  |  | Multivariate | 25 DG FLAIR | 1.005 | [0.957, 1.055] | 0.846 |
|  |  |  | Age | 1.015 | [0.966, 1.067] | 0.551 |
|  |  |  | Sex (M) | 2.353 | [0.787, 7.036] | 0.126 |
|  | BevCyto | Univariate | 25 DG FLAIR | 0.966 | [0.932, 1.002] | 0.062 |
|  |  | Multivariate | 25 DG FLAIR | 0.968 | [0.932, 1.006] | 0.099 |
|  |  |  | Age | 1.010 | [0.983, 1.037] | 0.482 |
|  |  |  | Sex (M) | 1.484 | [0.653, 3.372] | 0.345 |
